# Tracking scientific discovery of avian phylogenetic diversity over 250 years

**DOI:** 10.1098/rspb.2022.0088

**Published:** 2022-04-27

**Authors:** Deon Lum, Frank E. Rheindt, Ryan A. Chisholm

**Affiliations:** ^1^ Department of Earth and Environmental Sciences, University of Manchester, Oxford Road, Manchester M13 9PT, UK; ^2^ Department of Biological Sciences, National University of Singapore, 14 Science Drive 4, Singapore 117558, Singapore

**Keywords:** phylogenetic diversity, avian phylogenetic tree, bird discoveries, evolutionary distinctness

## Abstract

Estimating the total number of species on Earth has been a longstanding pursuit. Models project anywhere between 2 and 10 million species, and discovery of new species continues to the present day. Despite this, we hypothesized that our current knowledge of phylogenetic diversity (PD) may be almost complete because new discoveries may be less phylogenetically distinct than past discoveries. Focusing on birds, which are well studied, we generated a robust phylogenetic tree for most extant species by combining existing published trees and calculated each discovery's marginal contribution to known PD since the first formal species descriptions in 1758. We found that PD contributions began to plateau in the early 1900s, about half a century earlier than species richness. Relative contributions of each phylogenetic order to known PD shifted over the first 150 years, with a growing contribution of the hyper-diverse perching birds (Passeriformes) in particular, but after the early 1900s this has remained relatively stable. Altogether, this suggests that our knowledge of the evolutionary history of extant birds is mostly complete, with few discoveries of high evolutionary novelty left to be made, and that conclusions of studies using avian phylogenies are likely to be robust to future species discoveries.

## 1. Introduction

Since the beginning of modern taxonomy in 1758, the number of the world's known species has increased several fold: from around 10 000 species detailed in the 10th revision of *Systema Naturae* [1,2] to now well over 2 million [3]. Still, new species continue to be discovered, even in well-studied taxa such as birds [4]. Just how much diversity is still out there waiting to be discovered?

Knowing how complete the discovery record is can shed light on the importance of species discoveries and may also influence conservation decisions. For such reasons, and also because of our intrinsic desire to know about life on Earth, there have been efforts to estimate the total number of species currently in existence. Erwin's famous estimate of 30 million tropical arthropod species aside [5], projections of total species richness have put this value somewhere as low as 2 million [6] or as high as 9 million [7]. If the higher bound is to be believed, it would mean that our current knowledge of life on Earth is but a fraction of the total diversity on the planet.

These projections typically focus on the accumulation of species richness, but the completeness of the biodiversity discovery record may alternatively be assessed by considering how evolutionarily distinct a newly discovered species is relative to known species at the time of its discovery. If most newly described species today are highly evolutionarily distinct relative to known species upon discovery, it would suggest that there are still many evolutionarily novel species left to discover. Conversely, if most of these new species are evolutionarily closely related to ones described previously, it would suggest that our knowledge of the tree of life for the group under study is mostly complete. In practical terms, the perspective of evolutionary distinctness, rather than raw species richness, is increasingly the focus of conservation efforts (e.g. [8–10]).

A useful metric for assessing the evolutionary distinctiveness of a group of species is phylogenetic diversity (PD). While multiple measures of PD exist, we use it in the strictest sense: the sum of all branch lengths in a given set of species on a phylogenetic tree [11]. With this metric, we can assess the uniqueness of a new species discovery by considering the amount of PD that a species's discovery adds to the known tree at the time of discovery. We use the term ‘known PD’ in this paper to refer to PD currently known to science, whether for life on Earth as a whole or for just a particular taxonomic group. Where ambiguity is unlikely to arise, we abbreviate ‘known PD’ to just ‘PD’ for brevity, although we of course acknowledge that true PD is different from known PD (a new species's discovery increases the latter but does not alter the former).

Fine-scale phylogenetic knowledge continues to elude the scientific community for the vast majority of organisms, adding considerable obstacles to assessing the magnitude of PD that remains to be discovered and described. Birds are the best-known animal class on Earth: of the approximately 11 000 species known to science, the vast majority had already been described over 100 years ago [12], and phylogenetic information of some sort is available for the majority of the avian tree-of-life [13–16], rendering birds the organism of choice to investigate undiscovered PD.

Our objective in this paper was to explore how human knowledge of birds has accumulated over the past two and a half centuries of species discoveries through the lens of PD. To calculate how PD has changed with successive species discoveries, we needed a phylogenetic tree of all known birds and their dates of discovery. This allowed us to compute the PD added by each species at the time of its discovery, which is dependent on which other species were already known at this time and their evolutionary relationships to the new species. We used a published avian supertree [16] that contains nearly all known bird species and supplemented this with information from several more recent seminal trees published on the basis of genome-wide DNA [13–15,17]. Although discovery of new bird species continues to the present day [4], we hypothesized that most recently discovered species would have added low amounts of evolutionary information at the times of their discoveries and that the currently known phylogenetic tree of global birds would already be largely complete. Therefore, we expected to see a pronounced flattening of the PD accumulation curve over time relative to the species discovery curve.

## 2. Methods

### (a) Obtaining a complete avian species-level phylogeny

To obtain a phylogenetic tree of global birds, we first started with a supertree produced by Jetz *et al*. [16] in 2012 based on an accumulation of hundreds of phylogenetic studies then available, the vast majority of which were based on mitochondrial DNA (mtDNA). While this provided a fairly complete avian phylogeny at the time, recent advances (e.g. [13]) have suggested that higher-level relationships (i.e. orders and families) within the tree may be contentious. For example, the supertree places the Tinamiformes basal to all other palaeognaths whereas many more-recent phylogenetic studies based on genome-wide DNA have shown that the order is most likely nested within the clade [17,18]. To account for such inconsistencies, we used four other more-recent phylogenies, all based on genome-wide DNA and encompassing a considerable proportion of the avian tree-of-life, to rearrange and supplement the supertree [13–15,17]. Henceforth, we refer to each tree by its first author's name.

To generate a final tree that merges all five selected phylogenies, we relied on the order-level relationships in the Jarvis tree [12], the family-level relationships in the Prum tree [16], the family-level relationships of the Passeriformes in the Oliveros tree [14], and finally the species-level relationships within the Jetz tree [15]. The Harvey tree [13] was used to inform both the family- and species-level relationships within the entire suboscine clade.

We took the Jarvis tree, generated using whole-genome sequencing, as the best representation of the inter-order relationships for birds and rearranged the Prum tree, which we took as the best representation of bird families, using this information. This involved extracting all branches of the families of a given order in the Prum tree, rescaling the branch lengths to be the same as the total branch length of that order in Jarvis, and replacing the Jarvis branch with the rescaled one (electronic supplementary material, figure S1). However, there were a few palaeognath orders that were not represented by the Jarvis tree. For this, we inferred how the orders should be related based on Prum by assuming that the relative branch length of an unrepresented to a represented branch in Prum was the same in Jetz (electronic supplementary material, figure S2).

As the Prum tree does not include several families in the Passeriformes, we used the Oliveros tree for the family level relationships within the oscines and also replaced the entire suboscine clade with the Harvey tree, which contains nearly all known suboscines [14].

Finally, with this existing tree, we added the species-level relationships from Jetz (apart from the suboscines that were already added in with Harvey) using a similar process to the Jarvis–Prum merger (i.e. extracting, rescaling, and replacing the appropriate family level branch). We avoided the use of a consensus tree for the Jetz phylogeny, instead opting to use a sample of the first 1000 out of 10 000 of the trees provided on birdtree.org. This led to us having 1000 slightly different phylogenetic trees after the full merging process. Unlike our Jarvis–Prum merger described above, we noticed that some of the families in the Jetz phylogeny were not monophyletic, which could lead to issues when rescaling the branch lengths. For example, if the Jetz tree places all species of a hypothetical family in the same clade with a branch length of 5 units, but there is a single, erroneously placed, species outside of this block that has a larger branch length of 10 units, rescaling the branches in this configuration would assume a total branch length of 10 units as opposed to 5, thereby artificially shortening the branch lengths of all the other species in that family. To overcome this, we used a combination of expert opinion and smaller phylogenetic studies to identify species that were likely misplaced and causing some families to be non-monophyletic in the Jetz tree (electronic supplementary material, table S2). We ignored these species when extracting and rescaling branch lengths, and then attached them to the appropriate family as a basal polyphyly. While this inflates the branch lengths of these species (by setting each to the maximum possible value), it preserves the inter-species branch lengths of that family, making the tree as a whole more reliable.

For consistency between trees, we followed the taxonomy of Howard and Moore 4 (H&M4) [19,20], dropping any taxa in the tree that were not recognized as full species. This meant dropping all subspecies that may have been included in the various trees, or some newly discovered putative species in more recently generated trees. There were a handful of cases where we reassigned the species's family based on more recent knowledge of the species’s placement. For example, the Hylocitrea (*Hylocitrea bonensis*) was reassigned to its own monotypic family Hylocitreidae, as its placement in Hypocoliidae in H&M4 was uncertain [19]. Our final combined trees included 9711 of the 10 176 recognized species in H&M4.

### (b) Calculating known PD

Starting with the full phylogenetic tree, we first calculated the total known PD by summing up all branch lengths. Then, we removed the single most recently discovered species and recalculated the total known PD on the tree. The difference in PD between this new tree and the original one would be equal to the contribution of PD from the removed species. We repeated this for the second most recently discovered species, and so on. If more than one species was discovered in the same year, we randomized the removal order. We performed 100 randomizations of the order of removal and took the average PD contributed by a species across all permutations.

Here we take a species's discovery year to be the date of its formal description as given in H&M4, thereby accounting for the entirety of its discovery process from the collection of specimens in the field to the final recognition of the species in the scientific record, including any additional laboratory or bench work if necessary.

### (c) Analysing the accumulation of known PD

We assessed the accumulation of known PD both over time and over consecutive species discoveries. For both, we ran linear regression models to observe any changes in the trend of PD accumulation, with PD as the response variable and either time or species richness as the explanatory variable. Because a large number of species were described all at once in the first few years of Linnean taxonomy (beginning with Linnaeus’s *Systema Naturae*), we ran a separate linear regression that only considered species discovered from 1780 onwards. To improve the fit of the linear regression model, and also to ensure that predicted PD contributions cannot go below zero, we log-transformed the mean PD contributions from each species prior to model fitting.

To understand the effect of the discovery order on PD accumulation, we constructed a null model by rerunning the analysis after randomizing the sequence of species discovery. Here we randomized the discovery order for all species (as opposed to earlier when we randomized just those discovered in the same year) in each tree 100 times. This null model helped to highlight whether species discoveries over time have been random with respect to their position on the phylogeny or whether taxonomic efforts have been more selective, perhaps focussing on either more or less phylogenetically distinct species at different points in time.

In addition, we also looked at how PD accumulation changed when we reversed the species discovery order. This afforded a slightly different perspective to the null model and helped further highlight the differences between older and newer discoveries.

Finally, we looked at the change in PD in each order over time and species discoveries within that order to get a better idea of how known PD has changed within each order, and to see whether the individual clades have reached a plateau.

All analysis were done in R v. 4.0.4 [21]. Manipulation and analysis of phylogenetic trees was done with the ‘ape’ [22] and ‘phytools’ [23] packages. In the electronic supplementary material, we provide the merged phylogenetic trees as well as the R code and species list used to analyse them.

## 3. Results

### (a) Overall known PD accumulation

**Figure 1.**
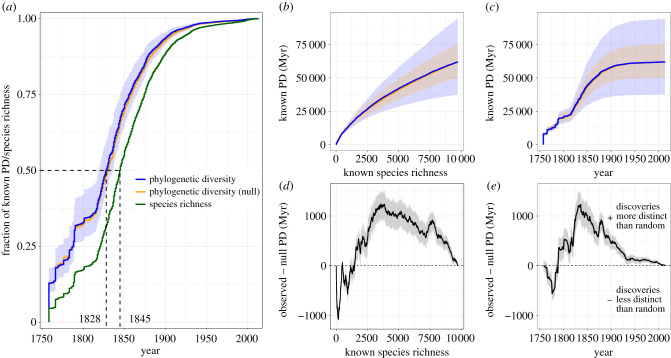
Accumulation of known PD and species richness in birds. (*a*) Cumulative fraction of present-day known PD (blue/orange) or species richness (green) discovered over the period of modern taxonomy. The dotted lines indicate when half of present-day PD or species richness had been discovered. (*b–c*) The accumulation of known PD over successive species discoveries (*b*) and over time (*c*). (*d*–*e*) The difference between the blue and orange lines in (*b*) and (*c*), respectively. In all figures, the orange line shows the known PD accumulation when the discovery order is randomized (i.e. the null model). Shaded areas indicate the 95% confidence intervals for known PD. (Online version in colour.)

Our updated avian tree included 9711 species and encapsulated over 60 billion years of evolutionary history. In the early years of bird species discovery, total PD accumulated quickly but then slowed down around the start of the 1800s before picking up again (figure 1*a*). By 1825, nearly half of today's known PD had been discovered. Known PD plateaued in the first half of the 1900s. Known species richness, in contrast, accumulated at a slower rate—the 50% milestone was reached around 25 years later in the 1840s and the figure only began to plateau around the 1950s (figure 1*a*); there has been a minor resurgence of species discoveries in recent decades. Despite this slight uptick of discoveries, however, the PD curve did not show the same pattern, suggesting that these newly found species have not made much of a contribution to PD. Performing a linear regression (electronic supplementary material, table S1) showed that the average PD contributed by a new species discovery decreased by on average 0.77% (SE = 0.02%) in each subsequent year, relative to the previous year's value. If we consider only species discovered from 1780 onwards, the decline was slightly lower at 0.63% (SE = 0.02%).

When considering known PD as a function of known species richness (figure 1*b*), we found that PD contributions from earlier species discoveries were higher than more-modern discoveries, as expected. Unlike the other curves, which were plotted as a function of time, we do not expect this to reach a plateau given that a new species has to contribute at least a minimum, non-zero amount of known PD to be placed on the tree. The average PD contributed by a new species discovery declined by an average of 0.013% (SE = 0.00031%) with each successive species discovery. When considering only species discovered from 1780, the decline was lower at about 0.011% (SE = 0.00036%).

Compared to what we observed, PD accumulation in the null model was higher for the first couple of thousand species descriptions (figure 1*b*), or up until around the year 1790 (figure 1*e*), but then fell below the observed values for most of the discovery process. This pattern points to the discovery process being non-random since the composition of discovered species has generally been more phylogenetically distinct than if the process were entirely random. We found a similar conclusion after reversing the discovery order: PD would have accumulated at a slower rate if recently discovered species were discovered initially (electronic supplementary material, figure S3).

### (b) Known PD accumulation by taxonomic order

**Figure 2.**
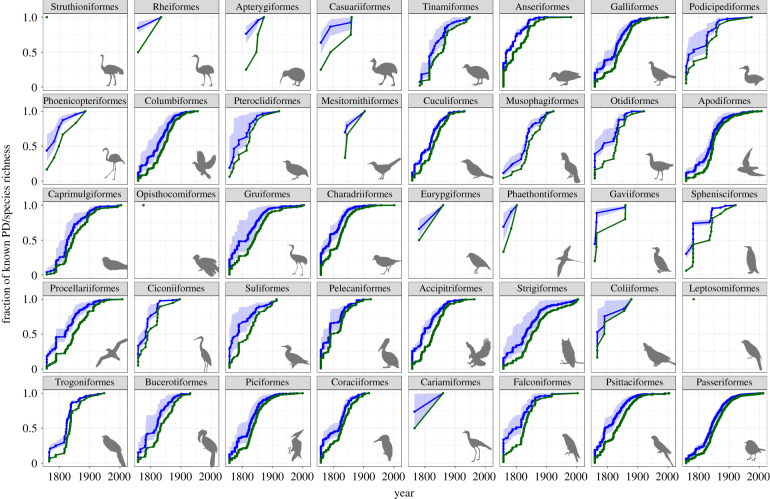
Fraction of known PD (in blue) and fraction of known species richness (in green) over time for each of the 40 avian orders. Each point corresponds to a species discovery. Shaded areas indicate the 95% CI for known PD. (Online version in colour.)

Similar to the trends in the overall data, we observed a steep increase in known PD accumulation in the initial starting years of taxonomic discovery for most avian orders. Most orders were well known by 1900, with the PD curve in many groups starting to plateau even before then (figure 2). For some (e.g. Accipitriformes, Anseriformes), the majority of the present-day PD had already been discovered before the 1800s. The plateauing of the PD accumulation curve was not as apparent for other groups, particularly some less speciose tropical orders (e.g. Casuariiformes, Coliiformes), likely due to the dearth of data points—any species discovered within one of these species-poor orders would have added a significant proportion of PD to that group, causing a sharp jump in the order's PD accumulation curve.

**Figure 3.**
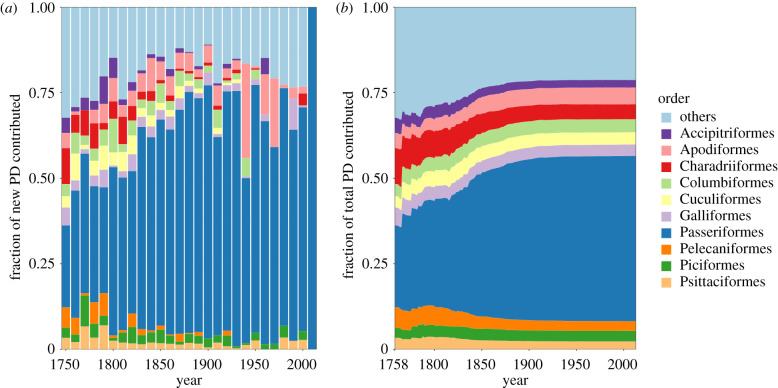
Relative fraction of known PD contributed by each order. (*a*) The fraction of new known PD contributed by each order within each decade. (*b*) The cumulative fraction of known PD contributed by each order over time. For visual clarity, only the highest 10 known PD contributing orders as of the present day are shown. Contributions from the remaining orders are shown as ‘others’. (Online version in colour.)

The hyper-diverse Passeriformes have contributed the most to new known PD in each decade since the beginning of the formal scientific discovery process, and this has been particularly true in recent decades (figure 3). The fraction of total known PD contributed by each order stabilized around the early 1900s and has been relatively constant since then. This, combined with the fact that most new discoveries have been from the Passeriformes, which have much shallower branches, suggests that the relative contributions of each order and the overall structure of the avian tree are unlikely to change with future species discoveries.

### (c) Distinctive species

**Figure 4.**
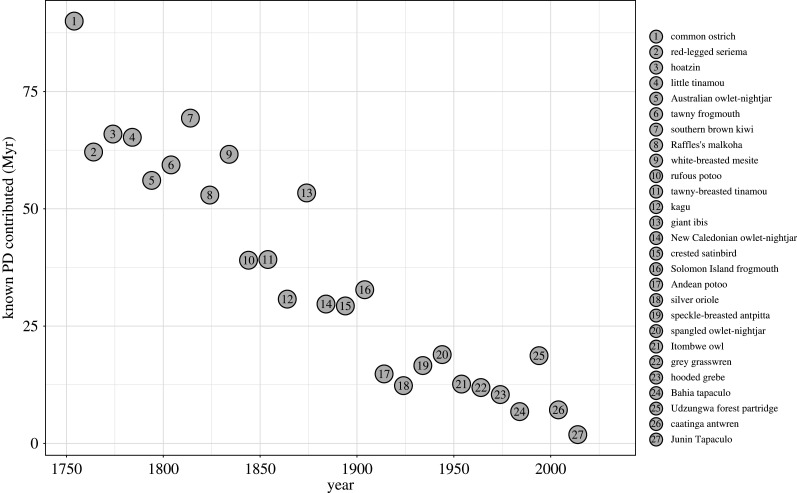
Contribution to known PD of the most distinctive species discovery (i.e. the species discovery making the greatest such contribution) in each decade. (Online version in colour.)

For each decade, we identified which newly discovered species was most distinctive, in that it led to the highest increment in known PD (figure 4). A handful of these distinctive species contributed large amounts of known PD simply because they were discovered early on (e.g. the little tinamou *Crypturellus soui*; data point 4 in figure 4), and would not have been so distinctive had they been discovered later. But many of them, such as the kagu (*Rhynochetos jubatus*; data point 12 in figure 4), are sole representatives of particular families or genera and would have been distinctive regardless of their time of discovery. The known PD contributed by the most distinctive species generally declined over successive decades: the discovery of the kagu in the 1860s contributed over 30.8 Myr to known PD, but this was only about half the average of previous decades' most distinctive species. And in our most recent decade (2010–2013) the most distinctive species was the Junín tapaculo (*Scytalopus gettyae*), which added a branch length of 1.9 Myr—the lowest contribution of any decade's most distinctive species and an order of magnitude lower than that of the kagu. There were some notable exceptions to the trend of declining contributions to known PD over time: for example, the remarkable discovery of the Udzungwa forest partridge (*Xenoperdix udzungwensis*; data point 25 in figure 4) from Tanzania in the 1990s added more known PD than any species discovered in the four decades prior.

## 4. Discussion

Our analysis suggests that current knowledge of the evolutionary history of extant birds on Earth is largely complete, with most—if not all—major clades having been discovered. Although undoubtedly some new species remain to be discovered, these are likely to be evolutionarily closely related to known species. The Inti Tanager (*Heliothraupis oneilli*), described from western Bolivia in late 2021 (and therefore not included in the present analysis), reinforces this point [24]. This genus-level discovery, when compared to discoveries in recent decades, is highly distinctive, adding a similar amount of PD to the curve as the Udzungwa Forest Partridge (*Xenoperdix udzungwensis*) in the 1980s. Yet, despite the species' relative uniqueness, it does not change the recent trajectory of a declining trend of PD contributions that we observed; it is unlikely that further new discoveries will add PD of similar magnitudes to discoveries in earlier centuries.

Our results imply that the conclusions of research relying on avian phylogenies (e.g. [25,26]) are likely to be robust to future discoveries of new species. Similarly, conservation decisions for birds are increasingly based on PD (e.g. [8,27,28]) and these are likely to be well informed by the currently known avian tree. By contrast, were we to apply our methods to a less well-studied species group, such as the insects [29], we would expect the tree to be relatively incomplete and the known PD accumulation curve to still be in the steeply increasing phase. This steeply increasing curve would introduce caveats into research conclusions based on PD, as can be appreciated if one compares the state of phylogenetic knowledge for various bird orders around 1850, when any assumptions on the global distribution of shorebird (Charadriiformes) diversity would already have been fairly accurate, while assumptions about swift (Apodiformes) diversity would have suffered from the fact that scientists had described only roughly half of the PD known in swifts today. For groups about which our knowledge is similarly limited today, conservation decisions based on PD may not be robust to new species discoveries. For example, when prioritizing areas for protection, the apparent low PD of some areas may reflect our present state of ignorance more than biological reality.

Our analysis was motivated by the common observation that PD is more reflective of the uniqueness of a new species discovery than other metrics, such as species richness. A further benefit of PD is that it is more robust to taxonomic lumping and splitting. With the advent of DNA sequencing techniques, it has become easier to identify cryptic species, leading to frequent changes in species numbers for many groups, for instance mammals [30] or birds [31]. While changes in species delimitation retroactively affect both the history of accumulation of species richness and known PD, they are likely to influence known PD proportionally less if taxonomic reassignments are of closely related species. In this way, measuring known diversity as PD helps circumvent the issue of our increasing ability to delimit species, and gives a picture of the state of knowledge of biodiversity that is unlikely to be greatly affected by future taxonomic revisions.

Although the known avian PD accumulation curve appears to be saturating (figure 1*a*), this does not undermine the importance of continued discovery efforts. For example, discoveries of new species that are closely related to existing ones (and thus contribute little to known PD) can help to illuminate the mechanisms that underlie speciation by identifying recently or presently diverging lineages. For example, some of the Malagasy warblers (Bernieridae) were discovered in the late 1900s, well after the known PD accumulation curve had begun to plateau. Yet discoveries of these species have allowed a deeper investigation into the concept of adaptive radiation for these island endemics [32]. Of course, such knowledge will also continue to be important for traditional conservation approaches that continue to focus on species diversity, rather than PD. For instance, the description of five new bird species [3] on the islands of Taliabu and Peleng off Sulawesi in 2020 led to a characterization of these islands as Endemic Bird Areas (EBA), a widely used metric to identify areas of high biological uniqueness around the world [33] that does not account for PD.

Further insights come from our analyses of accumulated known PD versus accumulated known species richness (figure 1*b*). The observed curve of known PD versus species richness exhibits a more rapid increase than predicted by a null model (in which species discovery dates were randomized) or a model in which the discovery order was reversed, suggesting that early taxonomists were not discovering species entirely at random, and were probably—consciously or not—targeting species that were relatively distinct from ones already known. More recent discoveries, in contrast, are not as distinct as we would expect were the discovery process entirely random (electronic supplementary material, figure S4). This result, while insightful, is not entirely surprising. Taxonomists may not intentionally sample in a non-random manner, but many species attributes, such as body size and geographical location, can influence a species’s probability of discovery [34,35]. It is probably the case that early collectors targeted morphologically more distinct species, which we now know tend to be also more phylogenetically distinct. At the same time, they lacked the tools to uncover and distinguish between morphologically similar species. In contrast to these early years of taxonomy, our understanding of the prevalence of cryptic diversity has grown [36] and methods to distinguish species beyond morphology—such as DNA sequencing and bioacoustics [37–39]—are now widely used.

Indeed, these curves of known PD versus species richness (figure 1*b*,*d*) are in some ways more revealing than the relationships of either of these variables to time (e.g. figure 1*a*,*c*,*e*), because they are less sensitive to the vicissitudes of science and politics that cause the activity of taxonomists to vary over time [40–43]. When looking at the known PD versus time curves, lulls in taxonomic activity can make it appear as though a plateau in species discoveries has been reached (e.g. the period 1758–1820 in figure 1*a*), wrongly implying that most species have been discovered. Similarly, in curves of species richness versus time, rapid increases due to sudden spurts of taxonomic effort can falsely suggest that there are many more species left to discover.

The curves of known PD versus species richness also inspire reflection on the nature of the modern taxonomic process. These curves cannot be expected to plateau as do the known PD versus time curves (figure 1*a*): this would imply that the PD contributed by newly discovered species approaches zero, which is inconsistent with how taxonomy is done. Instead, we would expect the gradient of these curves to tend to some fixed value that represents the minimum time for evolutionary divergence of two species against the backdrop of shifting species definitions over time. In the present study, for example, we found that this increment could be somewhere around 3 Myr (electronic supplementary material, figure S5) and seems to have stabilized somewhat—at least as compared to the early years of species discoveries—although this does not preclude particular species having lower or higher increments than this average. To the extent that the gradient of such a curve has not stabilized, this is partially due to continuing advances in methods for splitting species in the studied group.

We encourage further research constructing known PD accumulation curves for more taxonomic groups, to help pinpoint groups that are most poorly known (i.e. those whose curves exhibit least saturation) and should therefore be targeted with greater investment in molecular and field work. To apply our method to other groups, one would need a well-informed phylogenetic tree that contains the evolutionary history and divergences of all or most known species. Near-complete trees are available for some non-avian vertebrate groups [44–46] and efforts are being made to sequence all extant eukaryotes [47], so more detailed and reliable phylogenetic trees for these other taxa may be on the horizon. For other, poorly known taxa, application of our methods will await future large-scale genome projects aimed at completing the tree-of-life. The combined efforts of scientists studying PD in multiple taxonomic groups will eventually reveal how close we are to complete knowledge of the diversity of life on Earth.

## Data Availability

The datasets supporting this article have been uploaded as part of the electronic supplementary material. The data are provided in electronic supplementary material [48].
